# Rare food allergens 

**DOI:** 10.5414/ALX02135E

**Published:** 2021-01-12

**Authors:** Karin Hoffmann-Sommergruber

**Affiliations:** Department of Pathophysiology and Allergy Research, Medical University of Vienna, Vienna, Austria

**Keywords:** food allergens, IgE-mediated food allergies, novel foods, edible insects, allergen labelling

## Abstract

In food allergy, only a restricted number of protein families have been identified to contain allergenic proteins. These can be further grouped into major allergens, responsible for inducing allergic reactions in the majority of patients allergic to the food source, as compared to minor allergens only affecting a small number of food allergic patients. In addition, rare allergens have only been described for single cases so far. Rare allergens can derive from novel foods, including exotic varieties and foods not yet frequently consumed in certain regions. Also, new or modified processing strategies could induce a higher allergenicity in certain dietary proteins. And finally, low abundancy and/or low allergenic activity may also account for some rare allergens. For allergenic risk assessment, cross-reactivity of novel allergens with already known allergens is in place and facilitates the identification of potential new allergens, while de novo sensitization to yet undefined allergens can only be described retrospectively. This review presents some examples of recently identified rare allergens.

**German version published in Allergologie, Vol. 43, No. 7/2020, pp. 272-276**

## Introduction: Common food allergens 

A number of foods can induce symptoms ranging from mild to severe, life-threatening reactions. In general, a distinction needs to be made between food allergies and food intolerances. Food intolerances are due to an imbalance of enzymes needed for the digestion of dietary components and the respective substrate. For example, low levels or the absence of lactase, an enzyme that is required for the degradation of lactose, cause lactose intolerance-related symptoms. In the case of histamine intolerance, the lack of histamine oxidase, which is required for degrading histamine, induces symptoms [1]. 

In contrast, food allergies are immune-mediated reactions directed against harmless dietary proteins – food allergens. In type 1 allergies, specific immunoglobulin class E (IgE) antibodies are produced, which are causative for inducing symptoms upon re-exposure with the same or highly similar proteins. The prevalence of food allergies among adults is ~ 3 – 4% and in children up to 6% [2, 3]. 

Severity of an IgE-mediated food allergy ranges from mild, unpleasant symptoms, such as itching in the oral cavity, erythema, and sneezing, up to severe reactions, such as emesis, cramps, and asthma attacks, and finally life-threatening anaphylactic reactions. All these symptoms have an immediate onset. 

Therefore, a detailed diagnosis of food allergy and the identification of the causative allergen(s) is important – especially for allergic patients who have already experienced severe generalized reactions caused by food components. Moreover, for those patients at risk, the prescription of an adrenaline autoinjector is mandatory as well as the training of the patient and caregiver in how to use this device in case of emergency [4]. 

IgE-mediated food allergies can be grouped into primary and secondary food allergies. In primary food allergies, symptoms are caused by food consumption, while in secondary food allergies, allergens present in foods and sources of inhalation can induce allergic reactions. These cross-reactivities are induced when a patient is exposed by the same or highly similar protein present in pollens, fungal spores, dust particles, and foods. The route of sensitization usually starts with the inhalation exposure, followed by the food uptake. 

In childhood, the most frequent food allergen sources are milk and egg. Tree nuts and peanuts frequently induce allergic reactions in both adults and children. Fish and seafood are relevant allergen sources for adolescents and adults. Pollen-related food allergies based on primary sensitization to Bet v 1, the major birch pollen allergen, are frequently diagnosed in adolescents and adults. 

## What are rare food allergens? 

There are several ways to define “rare allergens”. One option is to include allergens present in exotic foods, which have only recently become available in certain geographic regions, such as fruits from Asia and Africa, new cultivars from fruits and vegetables, and new grain varieties. 

Another definition of rare allergens deals with a low number of reported cases of being allergic to proteins that are highly abundant in daily diet – proteins with a low allergenic potential. 

Furthermore, food processing can have an effect on food allergens, thus modulating the allergenic potential by either upregulating or reducing the immunologic activity. 

Finally, rare allergens can also be defined by their low expression rate in foods and thus significantly reduced risk of exposure, with only highly sensitized allergic patients inducing an immune response to low amounts of these allergens. 

## Edible insects – do they pose a risk for allergic patients? 

For a few years now, edible insects have become available on the European market. While this food source has been part of the daily diet in Asia for centuries, this is a novel food source in Europe. Moreover, edible insects are regarded as a highly valuable food due to the high protein content as well as efficient and low-cost culturing conditions. At present, food safety authorities classify them as “novel foods” and request an allergenic risk assessment. 

Some research groups have already addressed this issue and provided some relevant data with regard to patient groups with an increased risk for developing allergic reactions when consuming edible insects. 

Broekman et al. [5, 6] investigated, in a group of shrimp-allergic patients, reactivity towards edible insects and used mealworms as a model insect food. The patients from this cohort were all sensitized to tropomyosin, a food allergen present in seafood and snails. However, tropomyosin is also present in insects, and this protein shares high sequence similarity with the protein in seafood and molluscs, thus inducing allergic reactions due to IgE cross reactivity. Furthermore, sIgE directed against another cross-reactive allergen, arginine-kinase, present in crustaceans, also recognized this allergen in mealworm, and a novel IgE-binding protein, the “larval cuticle protein”, was detected. In addition to potential food allergies due to ingestion of insects, also the risk of developing inhalant allergies against edible insects was reported from breeders working at breeding farms [5, 6]. 

Looking at the studies of edible insects as a potential novel food allergen source, it becomes evident that the current methods applied for allergenic risk assessment are established and help to identify homologous/cross-reactive allergens present in novel foods [7]. However, when it comes to the identification of novel, so far unknown food allergens, this risk assessment strategy fails and needs additional tools. 

## Exotic meat should be avoided when suffering from chicken meat allergy! 

Exotic specialties are attractive, and many people enjoy novel foods. However, for food-allergic individuals, this may pose an additional and unexpected risk. Ballardini et al. [8] reported an anaphylactic reaction in a 13-year-old boy when consuming crocodile meat for the first time. After immediate treatment with rescue medication, the diagnostic follow-up confirmed that the patient was allergic to chicken and turkey meat. Since the first diagnosis at the age of 5 years, his family avoided both meat species meticulously. 

Further in vitro tests showed the presence of IgE antibodies directed against α-parvalbumin, present in both chicken and turkey meat samples. Both parvalbumins share the identical amino acid sequence. When looking for IgE cross-reactivity in the crocodile meat sample, a cross-reactive protein was detected and identified as α-parvalbumin, which shares 94% sequence similarity to the chicken homologue. 

So far, parvalbumins have been known as major food allergens in many fish species and have been described also from frog meat. However, these proteins belong to the group of β-parvalbumins. In the case of chicken meat allergy, α-parvalbumins are the causative food allergen. This type of food allergy is rather rare, and actual prevalence data are unknown to date. This European case report is the first one describing chicken meat allergy with clinical cross-reactivity to crocodile meat due to α-parvalbumins. 

## Lupine flour: Risk of cross-reactivity based on peanuts! 

Lupine (*Lupinus alba*) belongs to the botanical order Fabales. The beans are rich in proteins and fibers, low in fat and gluten free. Therefore, lupine represents a food source of high nutritional value. In Europe, more and more food products, such as pizza, pasta, and sauces, contain lupine flour replacing wheat and soy. 

Recently, Soller et al. [9] published a lupine allergy case report. A 10-year-old boy consumed ready-mix pancakes and immediately developed an anaphylactic reaction. The diagnostic tests confirmed an already-known peanut allergy. In addition, a positive prick test with lupine flour was identified. When contacting the company that produced the ready mix pancakes, a potential carry over with peanut proteins was ruled out. However, since lupine and peanut belong to the same botanical family, an allergic reaction to lupine proteins based on cross-reactivity was diagnosed. In a European study Moneret-Vautrin et al. [10] have already determined the potential risk of cross reactivity between peanut and lupine in peanut-allergic patients, between 4 and 28% in 1999. Based on this and related studies, a mandatory labelling of lupine as an allergenic ingredient came into force in Europe, Australia, and New Zealand. In contrast, this regulation was not implemented in Canada. The parents of the allergic boy approached the Canadian Food safety Authority, and together with the patient organization, Health Canada, initiated a campaign informing both the general public and patients about the potential risk of a cross reactivity between peanut and lupine food products [9]. 

## Allergy to beer – a rare disease! 

Compared to the daily consumption rate of beer worldwide, the risk of an allergic reaction is rather rare. However, there are some case reports on allergic reactions to beer ingredients. A UK study reported that a man developed an anaphylactic reaction to a special beer variety. In skin prick testing, he was positive for wheat, barley, and maize. The in vitro IgE tests provided positive results for Pru p 3 (nonspecific lipid transfer protein (nsLTP)) from peach. The following laboratory tests verified that the nsLTPs from wheat, barley, and maize were the causative allergen still present in this beer variety, while other varieties had much lower levels of these allergens [11]. 

Another case report from the United Kingdom highlighted the relevance of additional ingredients as allergens. A woman developed anaphylaxis upon consumption of a specific beer variety. When checking the ingredient list of this drink, coriander was listed as giving a special flavor to this beer. The diagnostic test in the patient verified that indeed coriander was the culprit allergen, while barley and hops tested negative [12]. 

In summary, allergic reactions to beer are indeed rare. The few case reports indicate that seed storage proteins, abundantly expressed in maize, barley, and hops are potential allergenic candidates. Especially nsLTPs are the most frequently identified food allergens. However, fermentation and production procedures are different and thus can reduce the final concentration of intact nsLTPs in beer varieties. Therefore, some beer varieties can be consumed by even highly sensitized allergic patients without developing reactions [13]. 

However, due to a vast variety of specific regional beer varieties, with different taste and addition of unconventional ingredients, which are not always labelled as such, a risk of developing an allergic reaction to hidden allergens is possible. Especially addition of spices represents a risk for the allergic consumer. 

## Conclusion 

Proteins present in exotic and novel foods that induce allergic reactions in only a small group of patients can be assigned to rare allergens. Another group of rare food allergens comprise proteins with a reduced allergenic potential and have thus been described in only a few cases as inducers, and those proteins are present in low quantities in food products. Finally, certain food processing could also up- or downregulate their allergenic activity. 

The current allergenic risk assessment strategy is based on a weight of evidence approach including a number of in vitro and in vivo methods to identify potential novel food allergens. The assessment is well developed to identify proteins with cross reactivity to already known allergens [7]. However, it is of limited value if yet unknown food allergens should be detected. For this de novo sensitization, additional methods need to be developed and applied to determine novel and also rare allergens. 

## Funding 

Karin Hoffmann-Sommergruber was supported by the Danube Allergy Research Cluster from the Government from Lower Austria (subproject #7). 

## Conflict of interest 

The author declares no conflict of interest in relation to this publication. 
